# Online follow-up of individuals with gastroesophageal reflux disease using a patient-reported outcomes instrument: results of an observational study

**DOI:** 10.1186/1471-230X-13-144

**Published:** 2013-10-01

**Authors:** Merel M Tielemans, Martijn GH van Oijen

**Affiliations:** 1 Department of Gastroenterology and Hepatology, Radboud University Medical Center, PO Box 9101, Internal code 455, Nijmegen 6500 HB, The Netherlands; 2 Department of Gastroenterology and Hepatology, Jeroen Bosch Hospital, PO Box 90153, ‘s-Hertogenbosch, ME 5200, The Netherlands; 3 Department of Gastroenterology and Hepatology, University Medical Center Utrecht, PO Box 85500, Internal code F02.618 Utrecht, GA 3508, The Netherlands; 4 Division of Digestive Diseases, David Geffen School of Medicine at UCLA, Los Angeles, CA, USA

**Keywords:** The internet, GERD, PPI, Healthcare visit, GerdQ

## Abstract

**Background:**

Many individuals with gastroesophageal reflux disease (GERD) never visit their general practitioner. Therefore, prospective data about GERD and its natural history in the general population are scarce. The aims of this study were to assess symptoms over time and consultation reasons in an Internet population with GERD.

**Methods:**

Visitors (18–79 years) to a GERD information website who completed the GerdQ self-assessment questionnaire were invited to participate. Follow-up GerdQ questionnaires were sent after 4, 12 and 24 weeks, and those who had a total GerdQ score ≥ 8 and responded to at least the baseline and 4-week questionnaires (within 2–7 weeks) were included in the analyses. Outcome in proton pump inhibitor (PPI) and non-PPI users was classified as symptom improvement, symptom persistence/stable symptoms, or symptom relapse according to GerdQ scores.

**Results:**

A total of 403 non-PPI users (mean age 48 years, 40% male) and 304 PPI users (mean age 51 years, 41% male) were included. After 24 weeks, symptom improvement was present in 66% of non-PPI users (45/68) and 8% of PPI users (6/73), while persisting symptoms were reported by 24% (16/68) and 89% (65/73) respectively (baseline symptoms did not influence outcome at 24 weeks). Fifty-five percent of PPI users (116/210) and 37% of non-PPI users (76/207) who intended to visit a healthcare practitioner, performed one or more healthcare visits in the interim. Most frequently reported reason for consultation was persistence of symptoms.

**Conclusions:**

GERD symptoms were persistent in the majority of PPI users during our 24-week follow-up, while almost two thirds of non-PPI users reported symptom improvement. Online follow-up of an Internet population with GERD is feasible.

## Background

Gastroesophageal reflux disease (GERD) is a frequent disorder with a prevalence in Western countries of around 10-20% [1-3]. As GERD is common in the middle-aged population, it is associated with decreased work productivity, including work absenteeism, leading to substantial indirect healthcare costs [4-7].

Despite the high burden of GERD on available healthcare resources, data about the natural course of GERD are scarce [8,9]. As ‘second best’, data from placebo groups included in randomized therapeutic trials can be evaluated to develop insight into the natural history of GERD. However, those studies are mainly performed in primary and secondary care, where only around 30% of individuals with GERD symptoms (range: 5 - 56%) ever present with their symptoms [10]. Consequently, many individuals that suffer from GERD are not considered for inclusion in those studies.

Use of the Internet is nowadays widespread and many individuals use this source for healthcare information [11-14]. A Dutch website with information about GERD was launched and website visitors could complete an online survey about symptoms and proton pump inhibitor (PPI) use. Reasons for visiting a general practitioner or to refrain from consultation were also asked. This model provides a unique opportunity to evaluate a population that has not yet entered the healthcare arena.

The aims of our study are: 1) to prospectively assess GERD symptoms online; 2) to study healthcare practitioner consultation patterns; and 3) to study underlying reasons for healthcare visits.

## Methods

### Study design and participants

The Dutch website http://www.maagzuur.nl (“maagzuur” is Dutch for “gastric acid”) contains information regarding GERD symptoms, possible causes, lifestyle advice, diagnostic options and treatment. In May 2008 the Dutch translation of the GerdQ self-assessment questionnaire (Table 1) was launched on this website and could be completed by all website visitors. After a preparatory period of 6 months, questionnaires completed between 5 December 2008 and 2 April 2009 could be included in this study. Follow-up GerdQ questionnaires were sent to all participants (aged 18–79 years) who had a baseline total GerdQ score ≥ 8 and agreed to be contacted again. Questionnaires were sent to eligible respondents after 4, 12 and 24 weeks after completion of the baseline questionnaire. Those who did not complete the first follow-up survey within 7 weeks were excluded to minimize variance. In case of duplicate GerdQ questionnaires entries -defined as: identical IP address, birth year and gender- only the first completed GerdQ questionnaire was taken into account. Respondents were regarded PPI users as they stated acid suppressive medication use. The remainder was classified as non-PPI users.

**Table 1:** GerdQ self-assessment questionnaire

	**Symptoms in the**	**Symptom presence**			
	**previous week**				
		**0 days**	**1 day**	**2-3 **	**4-7 **
				**days**	**days**
	**Question:**				
1.	How often did you have a burning feeling behind your breastbone (heartburn)?	0	1	2	3
2.	How often did you have stomach contents (liquid or food) moving upwards to your throat or mouth (regurgitation)?	0	1	2	3
3.	How often did you have a pain in the center of the upper stomach?	3	2	1	0
4.	How often did you have nausea?	3	2	1	0
5.	How often did you have difficulty getting a good night’s sleep because of your heartburn and/or regurgitation?	0	1	2	3
6.	How often did you take additional medication for your heartburn and/or regurgitation other than what the physician told you to take (such as Maalox)?	0	1	2	3

The Medical Ethical Committee of the Radboud University Nijmegen assessed the research proposal of this study and concluded that it could be waived for ethical review, as we did not gather information about participants from other sources (e.g. medical records) and data storage occurred in accordance with Dutch law. For this reason, we did not obtain written informed consent of all participants.

### GerdQ self-assessment questionnaire

The GerdQ is a short and validated self-assessment questionnaire that assesses presence of GERD and determines the impact of symptoms on patients’ daily lives [15-18]. The GerdQ comprises six questions reflecting symptoms in the previous 7 days, and has been developed with questions from the Reflux Disease Questionnaire (RDQ), the Gastrointestinal Symptom Rating Scale (GSRS), and the Gastrointestinal symptom Scale (GIS), which are all validated disease-specific questionnaires [19-21]. The first two questions (1 and 2) are positive predictors of GERD, and a higher score suggests a higher symptom frequency. Questions 3 and 4 address dyspeptic symptoms that lower the probability for GERD, i.e. they are negative predictors of GERD. The two final questions (5 and 6) assess the impact of GERD symptoms on peoples’ lives and are also positive predictors of GERD. The score on every question ranges from 0 to 3 for the four positive predictors of GERD (0 days is a score of ‘0’, 1 day is ‘1’, 2–3 days is ‘2’, 4–7 days is ‘3’, or in reversed order for the two negative predictors of GERD) (Table 1).

### Outcomes

Our primary outcome in non-PPI users was “symptom improvement”, which was defined as a GerdQ score < 8 if the respondent scored ≥ 8 on the previous questionnaire. “Stable symptoms” were defined as GerdQ score ≥ 8 at two subsequent completed questionnaires during follow-up. “Relapse” was defined as GerdQ ≥ 8, in case the previous GerdQ score had been < 8.

Our primary outcome in PPI users, “symptom improvement”, was defined as a maximum of one day per week either heartburn (question 1), regurgitation (question 2), sleep disturbance (question 5), or over-the-counter (OTC) acid suppressive medication use (question 6), all during the preceding week. Persistence of GERD symptoms in PPI users was defined as more than one day per week with either heartburn (question 1), regurgitation (question 2), sleep disturbance (question 5), or OTC acid suppressive medication use (question 6), during the preceding week. If respondents reported symptoms more than one day per week for at least 2 subsequent GerdQ questionnaires, they fulfilled the criteria for “persistent symptoms”. If the participant reported an increase in symptoms from a maximum of one day per week to at least two times per week, this was defined as “symptom relapse”.

### Statistical analysis

Data were analyzed with SPSS version 18.0. Baselines variables for respondents without PPI use and PPI users were assessed with descriptive statistics. Percentages of symptom improvement, stable symptoms, and relapse were assessed separately for PPI and non-PPI users and were calculated with chi-squared analysis or Fisher exact, whenever appropriate. If one of the follow-up questionnaires was missing, data were compared with the previous completed questionnaire (e.g. if Survey C was missing, data of Survey D and B were compared). Frequencies of heartburn, regurgitation, sleep disturbances and OTC acid suppressive medication use during follow-up were calculated with frequency tables in respondents without PPI use. Mean symptom frequency within individuals during follow-up was assessed by paired t-tests in non-PPI users. We analyzed respondents according to (non-) PPI use at baseline.

Respondents were asked at baseline whether they had intended to visit a healthcare practitioner. During follow-up we asked whether they had actually visited a healthcare practitioner. Reasons for consultation were assessed with closed questions and presented in frequency tables. If respondents performed more than one healthcare visit during follow-up, only reasons for the first visit were taken into account. In respondents that did not visit a healthcare provider during follow-up, reasons that were reported in the last completed questionnaire were included and depicted in frequency tables.

Associations between outcome at 24 weeks and GerdQ score at baseline and type of symptom at baseline were analyzed with chi-squared analyses. We also analyzed the percentage of respondents that started or stopped their PPI with descriptive statistics. For this analysis, we only took the first medication switch into account. A per protocol analysis was performed, including only those respondents who did not change their use or non-PPI use during the 24-week follow-up. A P-value of < 0.05 was considered to be statistically significant.

## Results

**Figure 1:**
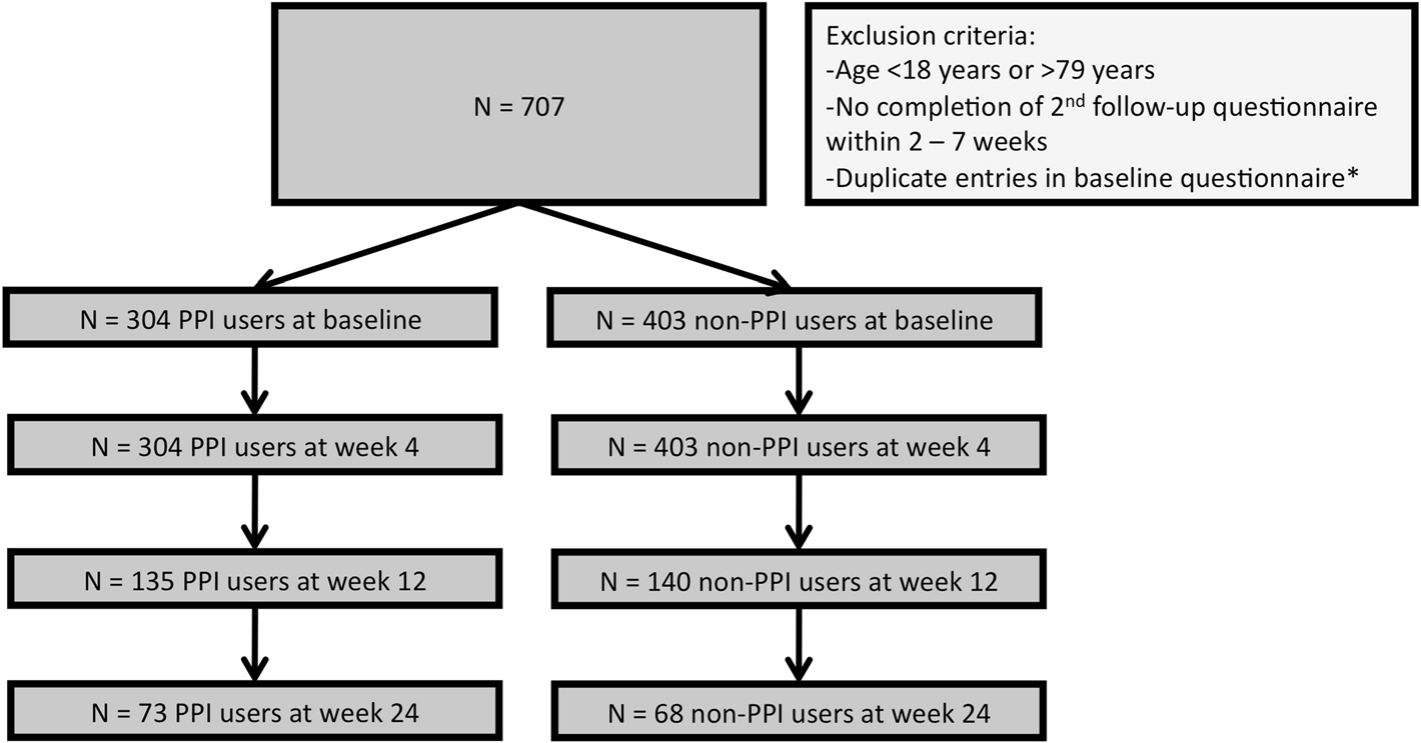
**Flowchart of participants.** *Identical IP-address, birth year and gender. PPI: proton pump inhibitor.

A total of 707 respondents met the predefined in- and exclusion criteria and completed the GerdQ between 5 December 2008 and 2 April 2009 (Figure 1). Forty-three percent of respondents (N = 304) reported PPI use, the remainder were classified as non-PPI users. Mean age of individuals without PPI use was 48 years (SD 13) and 40% was male. Mean age of PPI users was 51 years (SD 12) and 41% was male.

**Figure 2:**
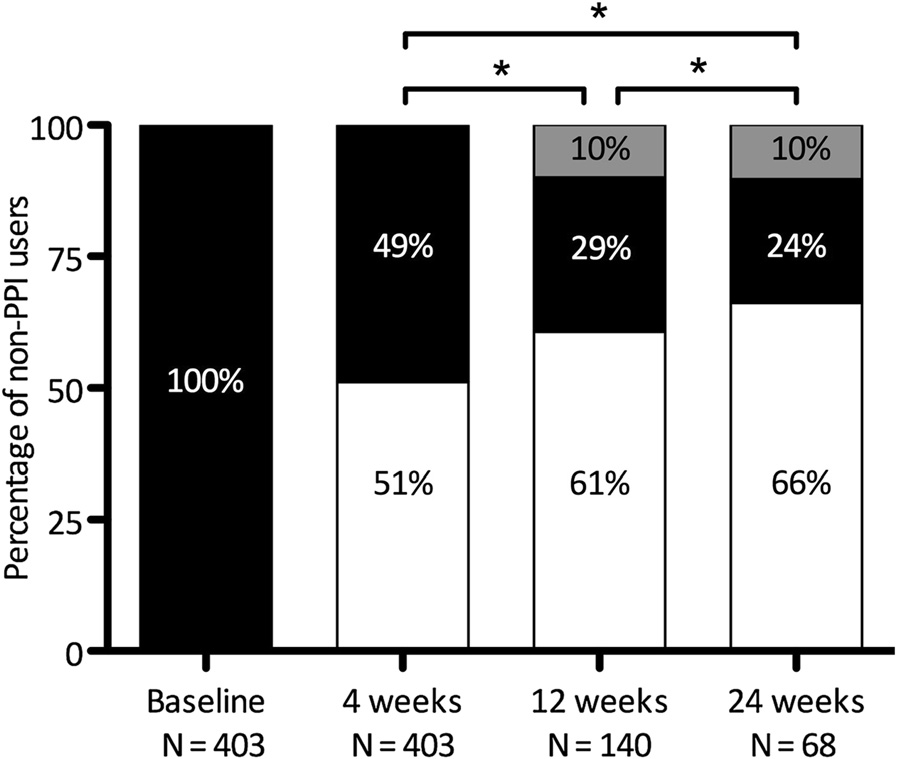
**Symptoms during follow-up in non-PPI users.** (White rectangle) GerdQ score < 8, i.e. no GERD according to GerdQ, or symptom improvement**. (Black rectangle) GerdQ score ≥ 8; i.e. presence of GERD symptoms or stable symptoms. (Grey rectangle) GerdQ score ≥ 8, if previous score < 8; i.e. relapse of symptoms**.** *P < 0.05. **See method section for definitions.

In the non-PPI using group, 68 respondents completed follow-up, of which symptom improvement was present in 45 respondents (66%) and relapse in 7 respondents (10%, Figure 2). Symptoms were persistent in the remaining 16 respondents (24%). In addition, we assessed 4 individual GerdQ questions during follow-up (Table 2). After 24 weeks, heartburn or regurgitation for a maximum of one day per week was reported by 44% and 81% of respondents without PPI use, respectively. Mean symptom frequencies of heartburn and regurgitation in non-PPI users significantly declined within individuals during follow-up from 2.21 at baseline to 1.43 at 24 weeks and from 1.20 to 0.77, respectively (both p < 0.01). Mean symptom frequencies of sleep disturbance and OTC use in non-PPI users declined from 1.52 to 1.20 (P = 0.30) and from 1.58 to 1.23 (p = 0.67), respectively.

**Table 2:** Presence of individual symptoms during follow-up in respondents without PPI use

	**Symptom frequency**			
	**0 days**	**1 day**	**2-3 days**	**4-7 days**
**Heartburn during the preceding week**				
**Baseline (%)**	25/403 (6.2)	46/403 (11.4)	152/403 (37.7)	180/403(44.7)
** 4 weeks (%)**	59/403 (14.6)	84/403 (20.8)	142/403 (35.2)	118/403 (29.3)
**12 weeks (%)**	23/140 (16.4)	40/140 (28.6)	41/140 (29.3)	36/140 (25.7)
**24 weeks (%)**	15/68 (22.1)	15/68 (22.1)	23/68 (33.8)	15/68 (22.1)
**Regurgitation during the preceding week**				
**Baseline (%)**	124/403 (30.8)	136/403 (33.7)	82/403 (20.3)	61/403 (19.4)
** 4 weeks (%)**	135/403 (33.5)	125/403 (31.0)	99/403 (24.6)	44/403 (10.9)
**12 weeks (%)**	61/140 (43.6)	40/140 (28.6)	26/140 (18.6)	13/140 (9.3)
**24 weeks (%)**	35/68 (51.5)	20/68 (29.4)	10/68 (14.7)	3/68 (4.4)
**Sleep disturbance during the preceding week**				
**Baseline (%)**	81/403 (20.1)	108/403 (26,8)	136/403 (33.7)	78/403 (19.4)
** 4 weeks (%)**	116/403 (28.8)	96/403 (23.8)	120/403 (29.8)	71/403 (17.6)
**12 weeks (%)**	49/140 (35.0)	36/140 (25.7)	34/140 (24.3)	21/140 (15.0)
**24 weeks (%)**	19/68 (27.9)	19/68 (27.9)	21/68 (30.9)	9/68 (13.2)
**OTC use during the preceding week**				
**Baseline (%)**	107/403 (26.6)	68/403 (16.9)	117/403 (29.0)	111/403 (27.5)
** 4 weeks (%)**	106/403 (26.3)	70/403 (17.4)	107/403 (26.6)	120/403 (29.8)
**12 weeks (%)**	39/140 (27.9)	30/140 (21.4)	33/140 (23.6)	38/140 (27.1)
**24 weeks (%)**	25/68 (36.8)	11/68 (16.2)	16/68 (23.5)	16/68 (25.3)

OTC: over-the-counter.

**Figure 3:**
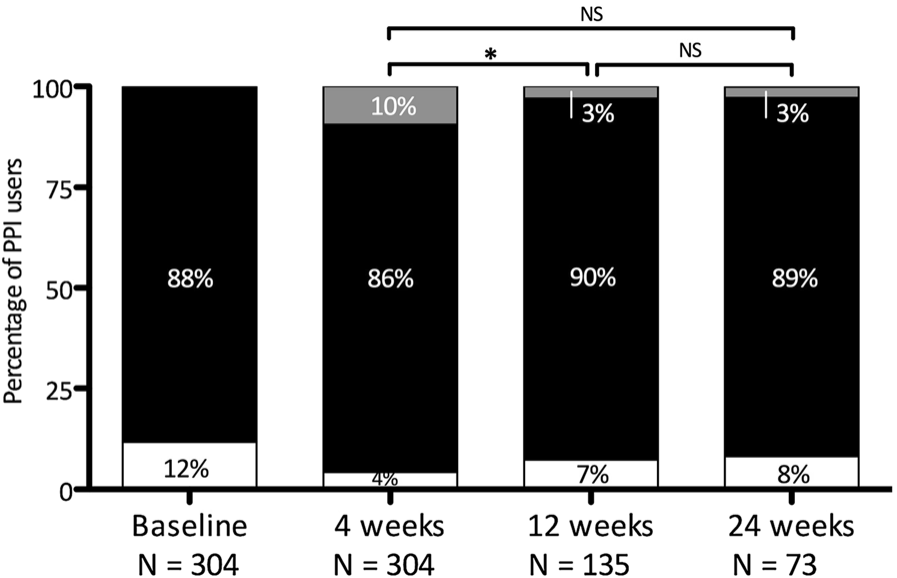
**Symptoms during follow-up in PPI users.** (White rectangle) Maximal 1 day per week symptoms**; i.e. “symptom improvement”. **(**Black rectangle) More than 1 day per week symptoms, i.e. Presence of GERD symptoms, or “persistent GERD symptoms”. (Grey rectangle) Relapse of symptoms. NS = not significant. *P < 0.05. **See method section for definitions.

In PPI users who completed follow-up (n = 73), 65 (89%) reported persistence of symptoms, 6 (8%) reported symptom improvement and 2 (3%) relapse of symptoms (Figure 3). Neither individual symptoms nor GerdQ scores at baseline were associated with symptom improvement at 24 weeks in respondents that did and did not use PPIs.

During follow-up, 22% started (n = 89/403) and 17% stopped PPI use (n = 53/304). If we excluded these individuals from our analyses, we found that 69% of individuals without PPI use reported symptom improvement (33/48) at 24 weeks in this ‘per protocol’ analysis. In PPI users, 2 participants (2/61; 3%) reported symptom improvement, and the majority (58/61; 95%) reported persistent symptoms.

### Healthcare consultation patterns

At baseline, 207 respondents without PPI use reported the intention to visit a healthcare provider. A total of 63 (30%) and 76 (37%) respondents who were planning to visit a physician had indeed visited a healthcare practitioner after 4 weeks and 24 weeks, respectively. A total of 210 PPI users intended this visit, and 94 respondents (45%) had visited a physician after 4 weeks. At the end of follow-up, 116 (55%) PPI users with the intention to visit a physician had actually done so.

The most reported reason to consult a healthcare provider was persistence of GERD symptoms, which was mentioned by 68% of non-PPI users and 73% of PPI users (Table 3). For non-PPI users, worries (44%) and impact on daily life (41%) were also frequently mentioned. In PPI users, impact on daily life (52%), and increased GERD symptom severity (40%) were frequently described reasons. The most reported reason to refrain from consultation was insufficient GERD symptom severity in non-PPI users (44%), and in PPI users (21%, Table 4).

**Table 3:** Reasons for consultation for GERD symptoms*

	**Non-PPI users**	**PPI users**
	**N = 95**	**N = 134**
GERD symptom persistence (%)	65 (68)	98 (73)
Increased GERD symptom severity (%)	34 (36)	54 (40)
No effect previous treatment (%)	8 (8)	49 (37)
Impact on daily life (%)	39 (41)	69 (52)
Someone else advised me to consult (%)	12 (13)	8 (6)
Information (%)	12 (13)	11 (8)
Worried (%)	42 (44)	49 (37)
Anxiety for serious cause (%)	19 (20)	40 (30)
Other reason (%)	2 (2)	4 (3)

*Respondents could report more than one reason.

PPI: proton pump inhibitor.

**Table 4:** Reasons not visiting healthcare provider for GERD symptoms*

	**Non-PPI users**	**PPI users**
	**N = 308**	**N = 170**
Expectation of decreasing GERD symptoms (%)	79 (26)	18 (11)
Insufficient GERD symptom severity (%)	136 (44)	35 (21)
Confidence in life style changes (%)	105 (34)	36 (21)
Over-the-counter medication use (%)	104 (34)	10 (6)
Do not want to take medication (%)	24 (8)	5 (3)
Afraid of diagnosis (%)	9 (3)	1 (1)
Do not rely on the doctor (%)	6 (2)	7 (4)
Do not make time to visit healthcare provider (%)	71 (23)	16 (9)
Other reason (%)	27 (9)	80 (47)
I do not know anymore (%)	6(2)	9 (5)

*Respondents could report more than one reason.

PPI: proton pump inhibitor.

## Discussion

We found that 66% of the individuals without PPI use reported symptom improvement at the end of follow-up at 24 weeks. In contrast, only 8% of PPI users reported symptom improvement at the end of follow-up and 89% of PPI users reported persistent symptoms.

Limited data are available about long-term effectiveness of PPI therapy in GERD. Short-term studies conclude that 17-45% of patients with GERD do not respond adequately to PPI therapy [22]. Symptom severity was comparable or had improved in the majority of patients after five years in the proGERD study [23]. However, patients included in this proGERD study were recruited from secondary care, whilst our population was not selected by physicians and we did not apply strict inclusion and exclusion criteria. Our population probably also contains respondents with functional upper gastrointestinal symptoms who are less likely to respond to PPI therapy than those with GERD. These factors, in addition to selection bias, could have contributed to the very high rate of persistence of symptoms in PPI users.

As we also focused on a different GERD population, namely Internet users with GERD symptoms without PPI use, we are not able to directly compare our results with others. As second best, we can use placebo responses in clinical trials. A meta-analysis in patients with GERD concluded that the average placebo response was 19% [24]. Follow-up of included studies was short with a maximum of 12 weeks. We found a higher percentage of symptom improvement at 4 and 12 weeks. This can be explained by the inclusion of patients with more severe symptoms in clinical trials and by the definition we used for symptom improvement.

Because many individuals with GERD symptoms refrain from consultations, it is interesting to assess underlying reasons for the decision to visit or not. In a survey among GERD patients in primary care, 52% mentioned that “symptoms too uncomfortable to bear” was the main reason for consultation [25]. The most frequently reported reason for consultation in our study was persistence of GERD symptoms (68% in non-PPI users, 73% in PPI users). Worries about their symptoms were reported by 44%% of non-PPI users and 37% of PPI users. Fear is frequently thought to be one of the most important reasons for seeking help, but we were not able to confirm this assumption.

We used the 6-item GerdQ self-assessment questionnaire for follow-up of GERD. The GerdQ appears to be a very promising tool to assess GERD symptoms in a structured, easy way and it is increasingly being used in clinical practice. A recently published study compared a treatment-algorithm based on the GerdQ with common practice of upper endoscopy and if indicated, pH metry in patients with GERD symptoms without any alarm signs. Use of the GerdQ approach was associated with a decrease in healthcare expenses, but had a comparable efficacy [16].

We believe that our data adds to the total, diverse population of individuals with GERD, of which only a minority visits healthcare practitioners. We were able to demonstrate how GERD symptoms evolve on and off PPI treatment. However, including respondents online is associated with limitations, most importantly selection bias. We faced a high dropout rate, probably related to the noncommittal attitude of an online questionnaire and the fact that we asked respondents to complete a total of 4 questionnaires during follow-up. We also do not have additional information about the medical history, comorbidity and reports of any additional investigations, such as upper endoscopy. We therefore cannot exclude that we included individuals with other diagnoses than GERD, or with concomitant diseases in addition to GERD. Another limitation is that we did not question the type and dose of PPI and the duration of use.

### Implications

Our study has implications for clinical practice. We have shown that it is feasible to use the GerdQ self-assessment questionnaire in PPI users to assess response to acid suppressive therapy over time. We observed that two thirds of non-PPI users had symptom improvement after 24 weeks. This supports the guidelines wherein first treatment step is lifestyle advice [26,27]. Effectiveness of lifestyle interventions has never been systematically studied, but in specific individuals these measures appear to be successful. In addition, our respondents reported confidence in lifestyle interventions (Table 4). When symptoms persist after lifestyle interventions, PPIs can be prescribed.

Our unique approach of online incorporation and follow-up of individuals with GERD demonstrates that the Internet can be used to trace individuals with specific symptoms and the follow-up via the Internet can be used as complementary method to the traditional routes. The communication in our study was one directional, but we will foresee an increase in online health platforms with direct patient-physician communication by e-mail, blog, or message services.

## Conclusions

We found in our 24-week follow-up study via the Internet, that more than half of the respondents without PPI use reported symptom improvement. However, more than 90% of PPI users reported persistence of symptoms. The most frequently mentioned reason for healthcare visits was persistence of symptoms. Based on our results, we support the use of the GerdQ to assess GERD symptoms and we agree with current guidelines that PPI prescription is not the first treatment step when patients present with symptoms suggestive of GERD. We have shown that online follow-up of an Internet population with GERD is feasible.

## Abbreviations

GERD: Gastroesophageal reflux disease; OTC: Over-the-counter; PPI: Proton pump inhibitor.

## Competing interests

MM Tielemans has no conflicts of interest. MGH van Oijen has received grant support from AstraZeneca and Janssen, and has served as a consultant for AstraZeneca and Pfizer. Sponsor: AstraZeneca BV, the Netherlands, financially supported the website: http://www.maagzuur.nl. The sponsor did not have any influence on the analysis, writing, and conclusions of the article.

## Authors’ contributions

MGHvO designed the study. MMT and MGHvO performed statistical analyses. MMT and MGHvO drafted the first and subsequent versions of the manuscript and both read and approved the final manuscript.

## Pre-publication history

The pre-publication history for this paper can be accessed here:

http://www.biomedcentral.com/1471-230X/13/144/prepub
